# Sour grapes and sweet victories: How actions shape preferences

**DOI:** 10.1371/journal.pcbi.1006499

**Published:** 2019-01-07

**Authors:** Fabien Vinckier, Lionel Rigoux, Irma T. Kurniawan, Chen Hu, Sacha Bourgeois-Gironde, Jean Daunizeau, Mathias Pessiglione

**Affiliations:** 1 Motivation, Brain & Behavior (MBB) lab, Institut du Cerveau et de la Moelle épinière (ICM), Hôpital de la Pitié-Salpêtrière, Paris, France; 2 Inserm Unit 1127, CNRS Unit 7225, Université Pierre et Marie Curie (UPMC–Paris) Paris, France; 3 Department of Psychiatry, Service Hospitalo-Universitaire, Centre Hospitalier Sainte-Anne, Paris, France; 4 Université Paris Descartes, Sorbonne Paris Cité, INSERM UMR S894, Paris, France; 5 Translational Neurocircuitry Group, Max Planck Institute for Metabolism Research, Cologne, Germany; 6 Translational Neuromodeling Unit, Institute for Biomedical Engineering, University of Zurich and ETH Zurich, Zurich, Switzerland; 7 Laboratory for Social and Neural Systems Research, Department of Economics, University of Zurich, Zurich, Switzerland; 8 Laboratoire d'Économie Mathématique et de Microéconomie Appliquée (LEMMA), Université Panthéon-Assas, Paris, France; 9 Institut Jean-Nicod (IJN), CNRS UMR 8129, Ecole Normale Supérieure, Paris, France; Harvard University, UNITED STATES

## Abstract

Classical decision theory postulates that choices proceed from subjective values assigned to the probable outcomes of alternative actions. Some authors have argued that opposite causality should also be envisaged, with choices influencing subsequent values expressed in desirability ratings. The idea is that agents may increase their ratings of items that they have chosen in the first place, which has been typically explained by the need to reduce cognitive dissonance. However, evidence in favor of this reverse causality has been the topic of intense debates that have not reached consensus so far. Here, we take a novel approach using Bayesian techniques to compare models in which choices arise from stable (but noisy) underlying values (one-way causality) versus models in which values are in turn influenced by choices (two-way causality). Moreover, we examined whether in addition to choices, other components of previous actions, such as the effort invested and the eventual action outcome (success or failure), could also impact subsequent values. Finally, we assessed whether the putative changes in values were only expressed in explicit ratings, or whether they would also affect other value-related behaviors such as subsequent choices. Behavioral data were obtained from healthy participants in a rating-choice-rating-choice-rating paradigm, where the choice task involves deciding whether or not to exert a given physical effort to obtain a particular food item. Bayesian selection favored two-way causality models, where changes in value due to previous actions affected subsequent ratings, choices and action outcomes. Altogether, these findings may help explain how values and actions drift when several decisions are made successively, hence highlighting some shortcomings of classical decision theory.

## Author summary

The standard way to explain decisions is the so-called valuation/selection model, which includes 1) a value function that calculates desirability for every possible outcome of alternative actions and 2) a choice function that integrates outcome values and generates selection probability for every action. In this classical view, choices are therefore determined (in a probabilistic sense) by hidden values. However, some authors have argued that causality could also be reversed, meaning that values may in turn be influenced by choices. Yet existing demonstrations of reverse causality have been criticized because pseudo-effects may arise from statistical artifacts. Here, we take a novel computational approach that directly compares models with and without the existence of reverse causality, on the basis of behavioral data obtained from volunteers in a new task. The winning model is a generalization of the reverse causality hypothesis, showing that people tend to like more the items that they previously chose to pursue, and even more if they did obtain these items. These effects were manifest not only in desirability ratings but also in subsequent actions, showing that value changes were more profound than just verbal statements. Altogether, our results invite reconsideration of decision theory, showing that actions are not neutral to the values driving them, hence suggesting that the history of actions should be taken into account.

## Introduction

Classical decision-making theory states that when facing a choice, agents consider the cost attached to potential actions and the value of their expected outcomes, and select the option that gives the maximal net benefit [1–5]. In this classical view, values are subjective estimates of anticipated outcomes that drive action selection. However, an opposite perspective has been suggested where the reverse inference is made [6]: agents may infer values from the observation of their own behavior. The general logic is: “I have engaged this action in order to get that outcome, therefore this is how much I like that outcome”.

This reversed logic has been adopted in the well-known cognitive dissonance theory [7, 8]. According to this theory, people adjust their preferences in order to justify their actions, i.e. to reduce the dissonance between choices and likeability judgments. A paradigmatic task used to demonstrate cognitive dissonance is the free choice paradigm, in which participants rate the likeability of several items, then choose between items of similar ratings, and then rate these items again [9]. An impressive number of studies showed that relative to the first rating, the second rating is increased for chosen items and decreased for unchosen items. This effect has been termed choice-induced spread of preference [10–13], which implies reverse causality from actions to values.

However, this line of research has been recently challenged [14, 15]. Indeed, if both ratings and choices proceed from probabilistic distributions over internal value estimates, then the famous spread of preference (between chosen and unchosen items) can be observed without any causal determination from choice to rating. This statistical artifact is analogous to a “regression to the mean” (see Izuma & Murayama [16] for a review but also Alós-Ferrer & Shi [17] for an opposite view). The idea is that because value judgments are noisy, two different items A and B may be given a similar first rating by accident, even in presence of a preference (say A>B), i.e. a difference between means of probabilistic distributions. However, this difference should be expressed on average, so it is likely that during following choice, A will be selected over B, and that during next judgment, A will receive a higher rating than B. Therefore, choice can appear as predicting a spread of preference, even if values are stable (i.e., if probabilistic distributions are not affected by choice).

Chen & Risen suggested a clever way to assess this statistical artifact, with a control condition in which both ratings are performed before choices (RRC condition). This control condition has been implemented in several studies, beginning with Chen & Risen themselves, who confirmed that apparent spread of preference is observed even in the RRC condition [14, 15]. To assess whether choice-induced spread of preference can occur on top of the statistical artifact, the critical test is then to compare the control RRC condition to the classical free choice paradigm (RCR condition). Initial results were mixed [14, 15] but then several studies have validated the presence of an effect beyond the artifact, although not all experiments were conclusive [18, 19], [20, 21]. Another way to get around the artifact is to assign participants choices that they did not make, and therefore cannot incorporate information about underlying values. Even these fake choices were found to spread preferences [22–25]. Also, the spread of preference was more robust when choices were remembered at the time of the second rating [26, 27], providing evidence for a psychological mechanism (post-hoc justification of choice) above and beyond statistical regression to the mean.

The first aim of the present study was to address this question through a different approach: by comparing computational models in which values are stable versus models in which values can evolve as a function of choice. Using this computational approach, we assessed which model would best account for behavioral data acquired in a new version of the free choice paradigm, following the standard rating / choice / rating sequence.

The second aim of our study was to generalize the notion that actions determine values, beyond choice. In decision theory, expected values not only drive which action is selected but also with how much vigor it is performed [28–30]. Here again, a reverse inference could be postulated: agents may revise their values depending on the degree of effort they notice to have exerted. This may explain some everyday life phenomena that have been described as ‘fruit of labor’ or ‘effort justification’ or ‘contrast effect’: for example a beautiful landscape would be even more enjoyable after an exhausting walk [31]. In the laboratory, it has been shown that abstract shapes associated with high effort (to get a reward) are subsequently preferred to shapes associated with low effort (for the same reward), suggesting a positive impact of effort on value [32–34].

This logic may extend to the success or failure of the action engaged (whether the outcome was obtained or not), which can be taken as a proxy for the effort invested. Agents may devalue outcomes of actions that were chosen and initiated but not completed, whatever the reason. This post-failure devaluation may account for the case of the fox in the famous Aesop’s fable, who revised his judgment about the desirability of grapes that he failed to reach, leading to the saying “any fool can despise what he cannot get”. This story is often considered as a typical example of cognitive dissonance and received different psychological interpretations: pretending that grapes were sour could for instance attenuate frustration or temper the reputation of being a loser [13, 35, 36]. Surprisingly however, previous studies have not intended to disentangle the impact of choice, success vs. failure and actual effort expenditure on subsequent valuation. To do so, we implemented choice as a decision about whether to perform an effortful action for a particular reward, which is why the choice task is thereafter denoted effort task. The required effort was varied such that participants sometimes failed to complete the action. This design therefore enabled assessing the effects of three action-related variables (not only choice but also effort and success) on outcome value.

The third aim of our study was to determine whether choice only impacts declarative judgments or all value-based decisions. We previously showed that rating, effort and choice tasks elicited the same value function [37, 38]. However, many would argue that to faithfully reflect preferences, behavioral responses must bear consequences. Otherwise, if there is no cost in doing so, subjects may fake their preference, notably for social reputation concerns. It is correct that in many paradigms (including ours), declarative judgments have no further consequence, while choice and effort have an impact on the outcome: they determine the reward that participants get in the end. In our case, the outcome was a food reward, which mattered to participants because they were actually hungry. In behavioral economics, choice and effort would thus be considered as "incentivized" and therefore more tightly linked to underlying values than declarative judgments. Indeed, evidence supporting cognitive dissonance theory in the free choice paradigm comes precisely from explicit ratings, leaving open the interpretation that participants just pretend having preferences that are more aligned to their choices than they truly are.

However, this does not imply that underlying internal values remain stable, as it might be difficult to maintain separate declarative judgments and internal values for a vast collection of items in the long run. Indeed, changes in (explicit) preferences have been shown to persist for one week [19] and up to three years [20] after choices were made. The question remains whether such long-lasting spread of preference corresponds to changes in the internal values driving other behavioral outputs than explicit ratings, such as choice and effort allocation. If the famous fox was presented in a follow-up story with the same grapes, would he try again? And would he try even harder? To answer these questions, we examined whether choices would affect not only subsequent ratings but also other value-based behaviors, such as subsequent choice or effort production. To do so, we extended the experimental task sequence to rating / effort / rating / effort / rating.

**Fig 1:**
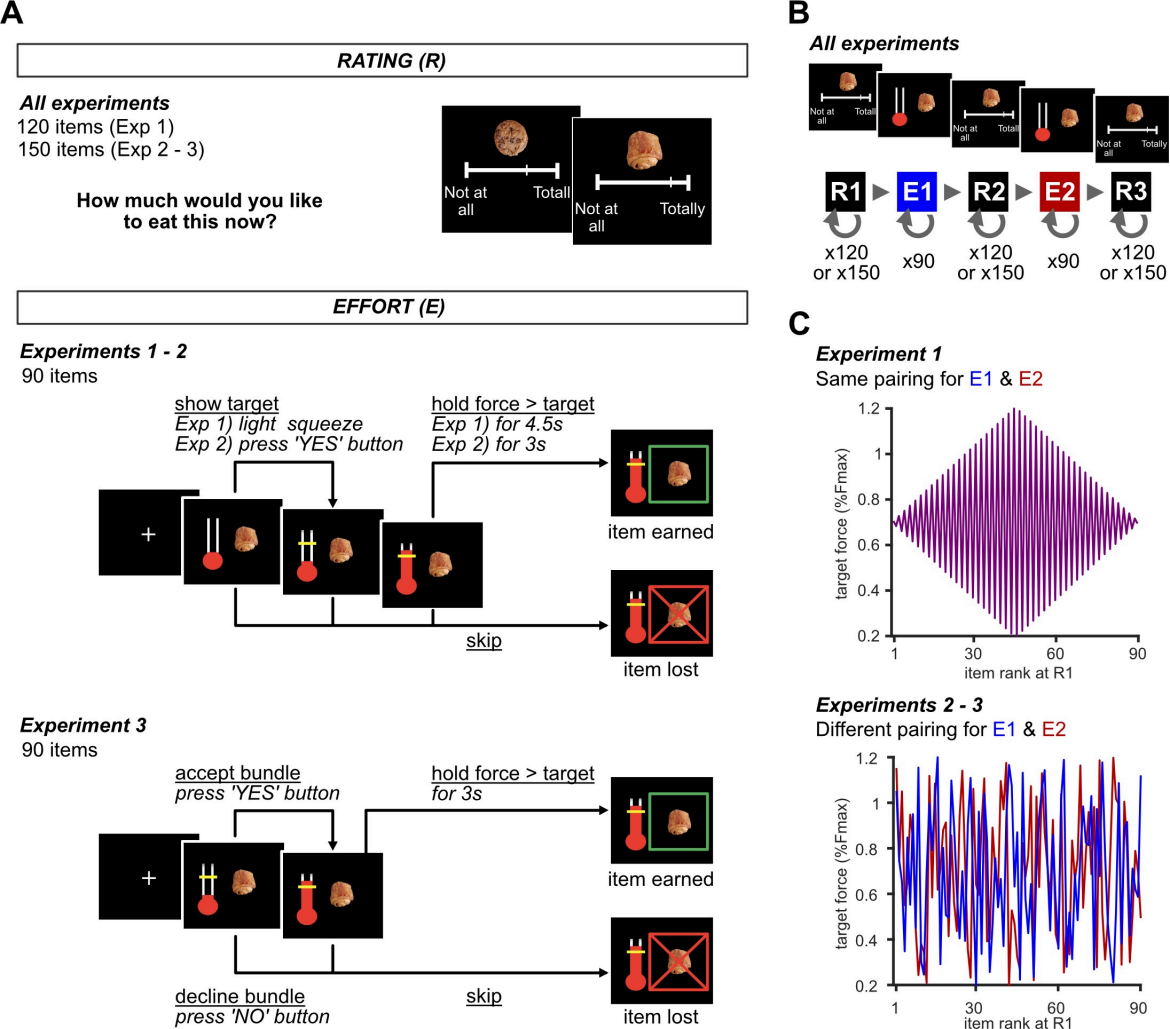
Experimental design. (A) Schematic representation of the behavioral tasks used in the three experiments (Exp 1–3). All tasks were self-paced. Screenshots of example trials are shown with time passing from left to right. Rating task (top): Subjects assigned likeability ratings to food items (120 in Exp 1; 150 in Exp 2 and 3), by moving a cursor along an analog visual scale. Food items were then ranked based on subject-specific ratings. We excluded the 15 items with highest and lowest ranking in all experiments, as well as 30 random items in Exp 2 and 3. Each of the remaining 90 items were paired with a specific target force level for the effort task (see illustration in part C for the two different pairings used in Exp 1 and Exp 2–3). Effort task (middle for Exp 1–2 and bottom for Exp 3): after fixation cross, a thermometer was presented next to a food item. Subjects could squeeze the handgrip to move the red bar up within the thermometer. In Exp 1, a light squeeze (5% *F*_*max*_) was sufficient to reveal the target force level required to earn the food item, but it was only obtained after force was maintained above target for at least 4.5s. In Exp 2, force level was only revealed if a ‘yes’ button was pressed, and food item was obtained after force was maintained above target for at least 3s. Otherwise, they pressed a ‘no’ button (equivalent to the ‘skip’ button) to reject the item without revealing the target force level. In Exp 3, both food item and the target level were presented simultaneously, and participants explicitly stated whether they would accept or decline (by pressing ‘yes’ or ‘no’ button, respectively) to perform the effort in order to earn the item. At any moment (in Exp 1), or after they had pressed ‘yes’ (in Exp 2 & 3), subjects could press a ‘skip’ button, in which case the item was lost, and the next trial began. A feedback screen indicated whether the item was successfully earned or lost, for a duration of 2 sec. (B) Sequence of behavioral tasks included in the entire experiment. The number of trials is indicated for both rating tasks and effort tasks. (C) Pairing of target force level with item ranking at R1 for 90 items (1^st^ is highest, 90^th^ lowest). The same pairing was used for all participants in Exp 1 (top), whereas pairing was pseudo-randomized in Exp 2 and 3 (bottom, only one participant is illustrated). In both pairing schemes, target force level and likeability rating were orthogonal.

To recapitulate, we designed a new free-choice paradigm that alternates rating tasks, in which participants make judgments about the desirability of food items, and effort tasks, in which they decide whether or not to produce handgrip force to obtain these items (Fig 1). Different force levels were assigned to different food items, orthogonally to desirability ratings. Some targets were beyond the maximal force that participants could reach, generating failure events analogous to the sour grape story. This design therefore dissociated three action-related dependent variables: choice (decision to engage effort exertion), effort (how much physical force was actually produced) and success (whether or not the outcome was eventually obtained). Note that the force produced, which was an observed behavior, was largely influenced by target force level, which was imposed by the experimenter. The three action-related variables were orthogonal, because success was only considered in accepted trials, and force in successful trials. We then examined the effects of these three action-related factors on all subsequent value-related behaviors: not only rating but also choice, effort, and success. In any case, there was no feedback in the sense that subjects never experienced outcomes, so change in values could only arise from action-related factors. The influence of past actions on subsequent behaviors was then assessed using computational modeling.

## Results

Three groups of participants (n1 = 18, n2 = 24, n3 = 24) were recruited to participate in slightly different versions of our new free-choice paradigm (Exp 1–3; see methods and Fig 1 for details). In all 3 experiments, rating tasks were alternated with choice tasks. In rating tasks, participants positioned a cursor on an analog scale to indicate the likeability of every food item. In the choice task, food items were paired with a given force level and participants decided between squeezing the grip above target for the imposed (constant) duration or simply proceeding to the next trial. Across experiments, the main noteworthy difference is the definition of choice (accept versus decline the offer). In Exp 1–2, choice was implicit: the criterion for acceptance was defined as participants squeezing the grip more than the minimal level required to get a food item (peak force > 20% F_max_). This is an indicator that participants did try to reach the target and get the item. Conversely, in Exp 3, choice was explicit: participants could accept or decline the offer (by pressing ‘yes’ and ‘no’ buttons) and then squeezed the grip to try and get the item. Other changes were introduced as additional controls. Notably, in Exp 2–3, 30 additional items were rated but not presented in the choice task, to assess the exposure effect (just seeing the items during choice could change their value). Also, the pairing of food items and force targets was constant in Exp 1 but redrawn in Exp 2–3, to avoid participants just repeating the same choice instead of evaluating the offer. Other differences were minor and meant to improve the design efficiency, although in the end, they did not produce much of an improvement.

### Sanity checks: Do ratings predict choice, effort and success?

**Fig 2:**
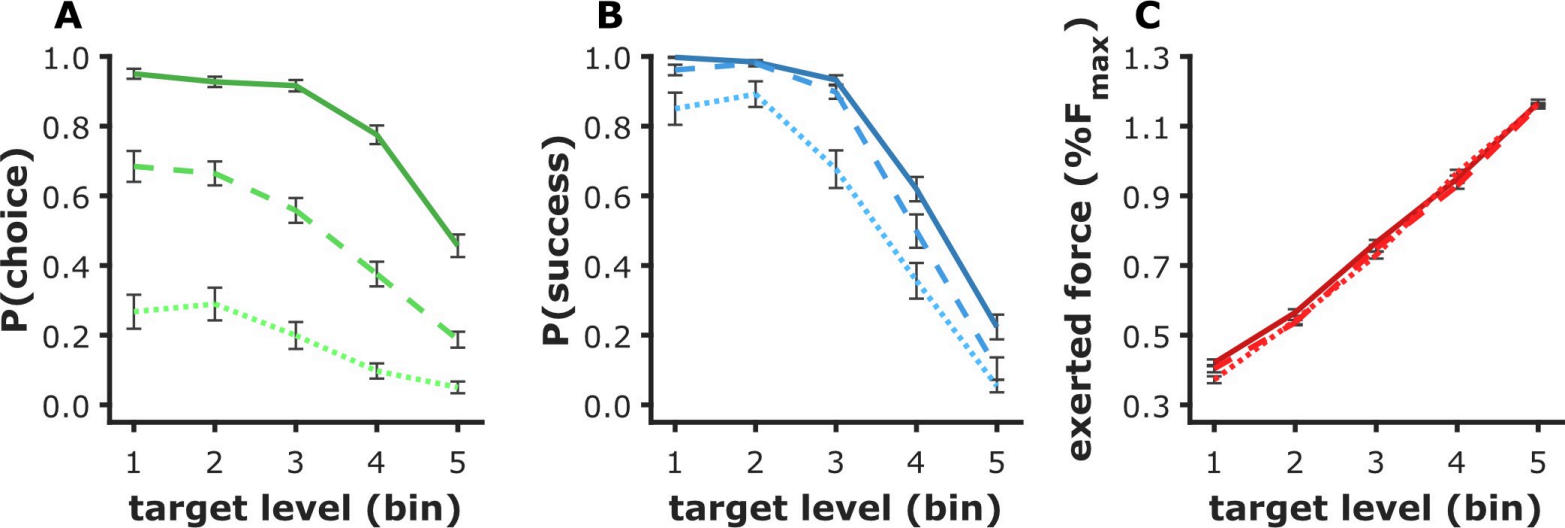
Action-related variables in the effort task. The three action-related dependent variables—choice (acceptance) rate (A), success rate (B), and exerted force (C) are shown as a function of target force level (imposed by the task). Only accepted trials were included in the analysis of success, and only successful trials in the analysis of force. Graphs show mean ± inter-subject SEM per force level. Darker colors and more continuous lines indicate bins (tertiles) of items with higher desirability ratings. Ratings were z-scored for every subject before binning.

We assumed that in all versions of the behavioral tasks, action-related variables (choice, success, and force produced) would result from a cost/benefit trade-off, the cost being here associated with physical effort, while the benefit varied with the value of items presented as potential outcomes [28]. We took target force level as a proxy for effort cost and likeability rating as a proxy for value. Therefore, we expected ratings and targets to predict all behavioral variables (choice, success, force) in the effort task. To test these predictions, for each participant, choice C, success S and force F were regressed across trials (i.e., across items) against target force level L and the desirability rating R given in the preceding rating session. The regression was run on the two effort tasks pooled together, but separately for each participant. Logistic regression models were used to account for choice and success (balanced accuracies of 0.78±0.01 and 0.82±0.01 respectively), while a linear regression model was used for force (R^2^ = 0.92±0.006). Individual regression estimates (betas) were then entered into group-level random-effect analyses. For success, only accepted trials were included (i.e., with force > 20% F_max_ for Exp 1–2, and yes response in Exp 3). For force, only successful trials (i.e., where required force was exerted) were included. As expected (Fig 2), ratings had a significant positive effect on subsequent choice, success, and exerted force (all p<0.01), denoting a higher motivation to win the food item. Conversely, target force level has significant negative effect on subsequent choice and success (both p<0.001), as well as a positive effect on exerted force (p<0.001). Note that the latter effect was trivially imposed by instructions: a successful trial means that exerted force reached target level. The negative effect on choice and success could be interpreted as a deterrent influence of anticipated effort cost (at the decision level) and experienced effort cost (at the execution level).

### Model-free analyses: Extending the free choice paradigm

As in usual free-choice paradigms, we expected post-choice ratings (relative to pre-choice ratings) to be higher for chosen items than for unchosen items. In other words, the choice variable should have a positive influence on the change from pre-choice to post-choice rating (the so-called spread of preference, hereafter denoted Δ-rating). We also tested the influence of the additional action-related factors that we integrated in our paradigm. According to the so-called ‘sour-grape’ effect, post-choice ratings should be higher for earned items (successful trials) than for lost items (failed trials), predicting again a positive influence of success on Δ-rating. Finally, according to the so-called ‘fruit of labor’ effect, the amount of effort expenditure should also have a positive influence on Δ-rating.

**Fig 3:**
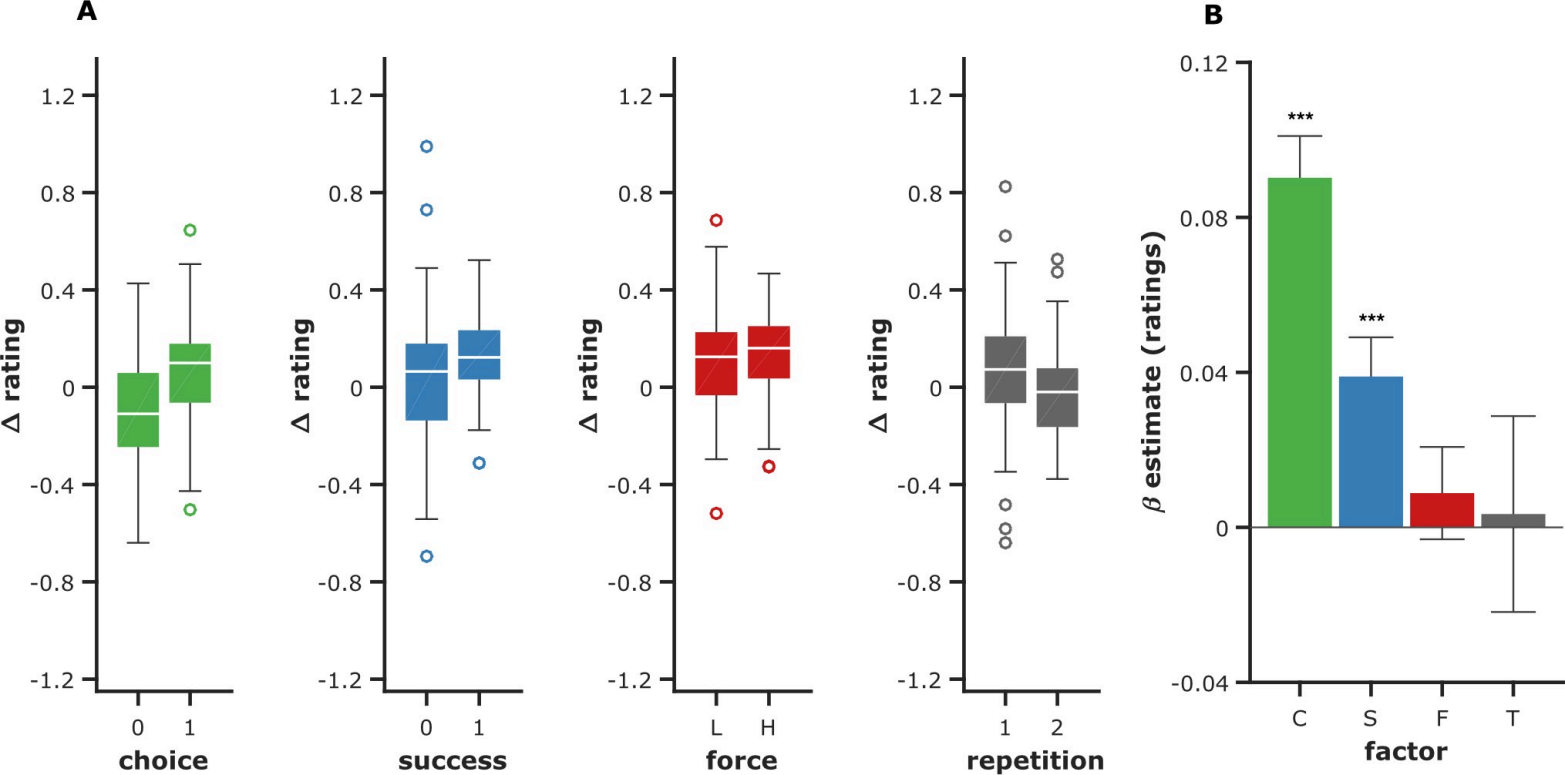
Influence of past actions on desirability ratings. (A) Left panels show the change in rating (R2-R1 and R3-R2 taken together) as a function of choice (0: unchosen, 1: chosen), success (0: unsuccessful, 1: successful), and exerted force (L: low, H: high, according to a median split). All trials were included to test the effect of choice, only accepted trials for the effect of success, and only successful trials for the effect of exerted force. The right most panel show the effect of task repetition (1 is R2-R1, 2 is R3-R2). White line: median, points are drawn as outliers if they are larger than Q3+1.5*(Q3-Q1) or smaller than Q1-1.5*(Q3-Q1), where Q1 and Q3 are the 25th and 75th percentiles, respectively. (B) Regression estimates corresponding to the choice (C), success (S), and force (F) factors, and to the intercept (T for time). Error bars represent inter-subject SEM. Stars and dots: * p<0.05; ** p<0.01; *** p<0.001.

To test these three predictions, we computed Δ-rating for each participant (ratings were z-scored within subjects before computing the difference), each item, and each repetition (i.e., R2—R1 and R3—R2). This Δ-rating was then linearly regressed against choice C, success S, and force F as predictors, pooling together the two repetitions. In order to orthogonalize regressors, all trials were z-scored within subjects for choice, while only chosen trials were z-scored for success (unchosen was coded 0) and only successful trials were z-scored for force (unsuccessful was coded 0). We also included a constant intercept term T that captured the effect of time. Individual regression estimates were then entered into group-level random-effect analyses (Fig 3B). The effects of choice and success were significantly positive (β_C_ = 0.09±0.01; β_S_ = 0.04±0.01, both p<0.001). This means that participants rated items higher after deciding to obtain these items, and beyond this effect, after successfully achieving the required force level. Note that even if we changed the definition of choice in Exp1/2 versus Exp3, and the pairing between ratings and targets in Exp1 versus Exp2/3 (see Fig 1 for details), the effect of choice on ratings was unchanged: it was significant in all experiments (Exp 1: β_C_ = 0.07, p = 0.008, Exp 2: β_C_ = 0.08, p<0.001, Exp 3: β_C_ = 0.11, p<0.001) and not significantly different between experiments (p = 0.389). Finally, there was no significant effect of exerted force (β_F_ = 0.009±0.01, p = 0.461) and the intercept was not different from 0 (T = 0.003±0.03, p = 0.892). Thus, we did not validate the hypothesis of a ‘fruit of labor’ effect, making items more valuable when they are obtained with more effort. Also, there was no evidence for a drift in rating, independent of task factors (i.e., when comparing R3-R2 to R2-R1, p = 0.484).

As all tasks were self-paced, one may wonder to what extent duration of exposure could be a confound, as difficult trials could take longer and thus prolong the exposition to related food items. In order to address this concern, we re-ran the same analysis but including cumulative duration of exposure (computed for each item at each rating) as an additional regressor. Critically, this regressor was not significant (p = 0.533) and the pattern of results was not modified.

Finally, we assessed whether the change in rating (i.e., Δ-rating) was driven by chosen items being rated higher, or unchosen items being rated lower, or both. We took advantage of the 30 items that were rated but randomly excluded from the effort task in Exp 2-3. Critically, Δ-rating for excluded items (+0.03) was in-between that of chosen items (+0.10) and unchosen items (-0.07), with a significant difference in both cases (both p< 0.001). Relative to excluded items, there was no difference in the magnitude of Δ-rating induced by acceptance and rejection (p = 0.194). Similarly, there was no difference in Δ-rating between excluded items and selected items (i.e., chosen and unchosen items taken together; p = 0.183).

Thus, model-free analyses provided evidence for an influence of choice and success on likeability ratings. However, these effects remain susceptible to the same statistical artifact (regression to the mean) as in the classical free choice paradigm. In addition, the complex interplay between experimental variables and behavioral measures was not properly assessed in the above model-free analyses that tested the different effects separately. To better assess whether the effect of past actions reflected an actual change in the underlying values, we therefore turned to a computational approach. As there was no evidence for a change in behavior across experiments, we pooled all participants in the following model-based analyses.

### Model-based analyses: Controlling for statistical artifacts

The general strategy was to formalize the null hypothesis, in which ratings are solely noisy observations of hidden values, and choice effects on ratings only statistical artifacts, and compare this null model to models that integrate the influence of past behaviors. The null model can be written as follows:

$$R_{i}=V_{i}+\varepsilon$$ (1)

where R_i_ and V_i_ are respectively the rating and value of item i, and ε is a Gaussian noise with variance $\sigma_{R}^{2}$. Thus, although the null hypothesis implies that values are strictly immutable, this null model predicts fluctuations in ratings due to the noise term. Fitting Eq (1) to actual ratings (i.e., estimating V_i_ and ε), therefore allows computing a likelihood that all changes in ratings are due to mere chance.

**Fig 4:**
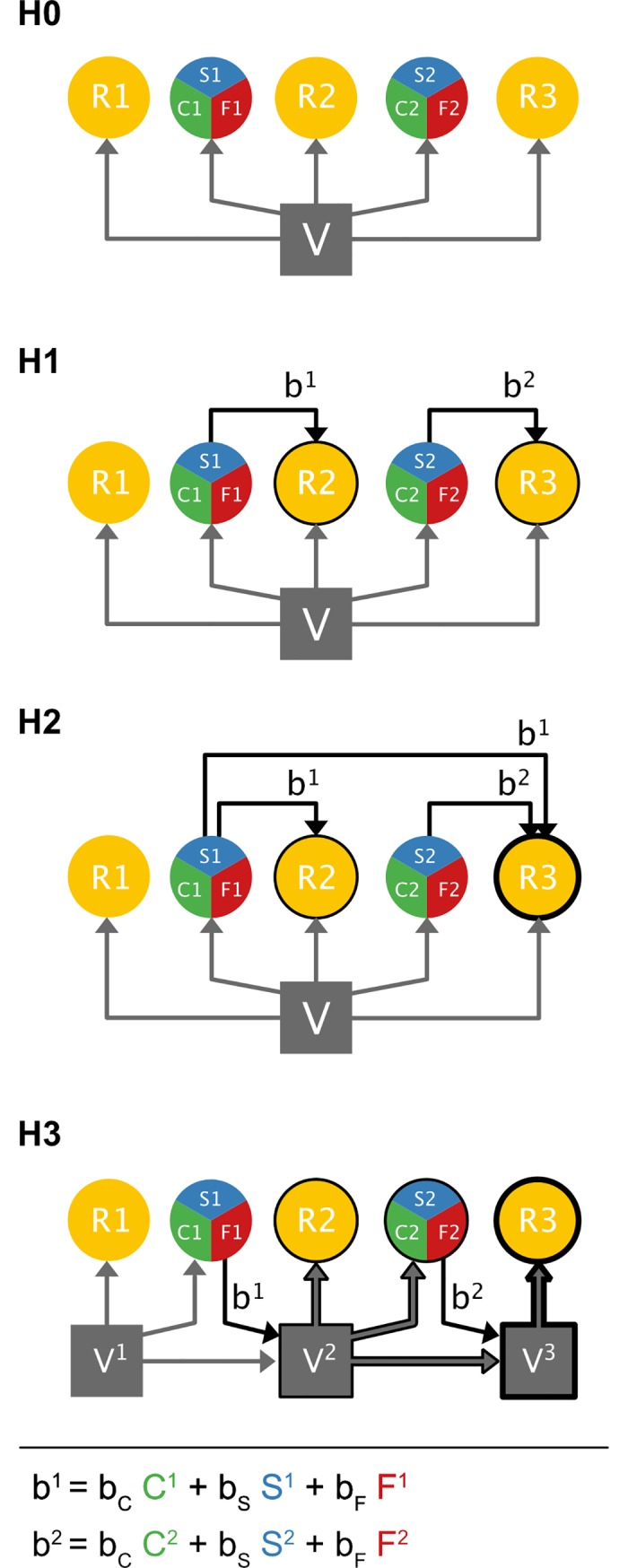
Model space. Schematic representation of the four hypotheses formalized in computational models. Colored circles represent behavioral responses in the rating and effort tasks: likeability rating (R) in yellow, choice (C) in green, success (S) in blue, and force (F) in red, the number indicating task session. Grey squares represent hidden values (V) of food items. Circles and squares are outlined in black, with line thickness indicating the cumulative impact of past actions. Black arrows represent the influence of past actions (b corresponding to the total bias, cumulated over action-related factors, as detailed in Eq 2). Grey arrows represent the influence of item values (V) on both declarative judgments (ratings) and actions (choice / success / force). The models differ by the extent of changes induced by action-related factors involved in the effort task: none in H0, restricted to next ratings in H1, extended to all subsequent ratings in H2, and generalized to all subsequent behaviors (both ratings and actions) in H3.

In the following, we detail a set of alternative models that could account for the influence of past actions on subsequent behaviors (see graphical illustration in Fig 4). In brief, on top of our null hypothesis (H0), we formalized three alternative scenarios with growing effects of action-related factors involved in effort tasks: in H1, only the next declarative judgment is affected (i.e., E1 could influence R2 but not R3 nor E2); in H2, all subsequent declarative judgments are affected but not the other actions (i.e., E1 could modulate R2 and R3 but not E2); in H3, all subsequent behaviors are modulated by previous actions (i.e. E1 could modulate R2, E2, and R3). The latter model corresponds to a change in underlying values that would affect all subsequent behaviors. For all three scenarios, each of the three action-related behaviors (choice, success, and force) could have or not a significant influence on the designated subsequent behaviors. All alternative models were assessed using Bayesian model comparison against the null model (Eq 1). This procedure allows us to derive statistical evidence for possible effects of past behaviors while controlling for the ‘regression to the mean’ confound. More precisely, any influence of past behaviors is only considered if it can explain subsequent ratings above and beyond the expected random fluctuations.

### Can actions affect post-hoc ratings?

In this section, we use Bayesian model selection to assess whether action-related factors affect subsequent ratings (model H1), as in the classical free-choice paradigm. We start by presenting the equations used to capture the direct bias that past actions may exert on next ratings.

As in model-free analyses, the effort task was decomposed into three behavioral variables: choice $C_{i}^{k}$, success $S_{i}^{k}$, and force $F_{i}^{k}$ where k denotes task session. They were orthogonalized to form three independent regressors, as follows:

$$C=\left\{ \begin{array}{cc} +1 & if\;trial\;is\;accepted\;(F>20\%\;F_{max}\;in\;Exp\;1-2;\;yes\;choice\;in\;Exp\;3)\\ -1 & otherwise\;(F<20\%\;F_{max}\;in\;Exp\;1-2;\;no\;choice\;in\;Exp.3) \end{array}\right.$$

$$S=\left\{ \begin{array}{cc} +1 & if\;item\;is\;successfully\;earned\;(F>L)\\ -1 & if\;trial\;is\;failed\;(20{\%\;F}_{max}<F<L)\\ 0 & otherwise\;(declined\;trial) \end{array}\right.$$

$$F=\left\{ \begin{array}{cc} z_{F} & z‑score\;of\;F\;if\;trial\;is\;successful\;(F>L)\\ 0 & otherwise\;(failed\;or\;declined\;trial,F<L) \end{array}\right.$$

Then, total bias $b_{i}^{k}$ induced by effort task k on the rating k+1 of item i can be formalized as the weighted sum of choice, success and force effects:

$$b_{i}^{k}=b_{C}C_{i}^{k}+b_{S}S_{i}^{k}+b_{F}F_{i}^{k}+b_{T}$$ (2)

The free parameters b_C_, b_s_, and b_F_ represent respectively the weight of choice, success, and exerted force. The parameter b_T_ captures the effect of time (i.e., repetition of ratings) and allows the model to capture non-specific trends like boredom (negative effects) or hunger (positive effect).

Finally, the hypothesis that these action-related variables could affect subsequent ratings can be written as:

$$R_{i}^{k+1}=V_{i}+b_{i}^{k}+\varepsilon$$ (3)

When all parameters b_C_, b_S_, b_F_, and b_T_ are (strictly) set to 0, Eq 3 is equivalent to Eq 1, which formalizes the null hypothesis H0: ratings are not affected by action-related variables and item values are solely determined by the constant free parameters V_i_. Conversely, if bias parameters b are allowed to differ from zero, then ratings will consistently change according to behavioral variables derived from the effort task, on top of spurious fluctuations driven by the noise term. Switching on or off the four bias parameters resulted in 16 different models. In order to test the possibility that actions have an impact on subsequent ratings (hypothesis H1), we grouped with H0 the model containing only an effect of task repetition (all b set to 0 except b_T_) and considered as belonging to H1 all other models that included at least one effect of choice, success, or force (at least one b ≠ 0 other than b_T_).

Note that Eq 2 is equivalent to the linear model that was used in the model-free analysis to assess the influence of action-related variables on subsequent ratings. However, because the Bayesian inference relies on the comparison to a prior assumption that all effects might be due to chance, any evidence for an influence of past actions in this model-based analysis is immune to the statistical artifact raised by Chen & Risen [14, 15].

A family-wise model comparison [39, 40] provided evidence that hypothesis H1 was far more plausible than H0 (Ef = 0.99, xp = 1). Furthermore, grouping models by action-related variables (choice, success, force) showed that all types of bias but force were significantly present in the population (see Table 1). This result does not imply that all biases had a consistent direction across subjects, since model inversion was performed at the individual level. To assess the consistency of effects across subjects, we estimated the amplitude of each bias at the individual level by computing the Bayesian Model Average (BMA) [41] of the posterior b parameters and entered them into group-level random-effect analyses (see Table 1). In accordance with model-free analyses, both choice and success effects were strongly positive (both p < 0.001). The effects of exerted force and task repetition were not sufficiently consistent across subjects to pass significance threshold.

**Table 1:** Effects of action-related factors on subsequent desirability ratings.

Family	Factor	Bayesian RFX (xp [Ef])	BMA Estimate (mean±sem)	p-value
H1	Choice (b_C_)	1.00 [0.99]	+ 3.1 ± 0.3	< 0.001 ***
	Success (b_S_)	1.00 [0.90]	+ 0.9 ± 0.1	< 0.001 ***
	Force (b_F_)	0.00 [0.05]	+ 0.2 ± 0.1	0.078
	Time (b_T_)	1.00 [0.99]	+ 0.1 ± 0.5	0.849

Results of group-level random-effect (RFX) Bayesian model selection (within a model space including both H0 and H1) are shown for the three action-related factors in different lines. Exceedance probability (xp) and expected frequency (Ef) are given for the family of models that includes the considered factor versus all other models. Bayesian Model Average of the corresponding bias (b) parameter is computed for each hypothesis separately and given as mean ± inter-subject SEM. Significance of the respective t-tests against 0 are noted

*** p<0.001, ** p<0.01, * p<0.05

This model-based analysis demonstrated that ratings were actually modulated by two action-related variables of the preceding effort task: choice and success. Indeed, higher ratings were assigned to items for which the trial was accepted (meaning that the subject decided to try and reach the target) and for which the item was earned (meaning that the subject succeeded in reaching the target). However, these effects may be short-lived and only affect ratings provided just after the effort task. In the next section, we examined whether the choice and success effects could extend to behaviors beyond the next immediate rating.

### Can actions affect distant ratings?

We first assessed whether actions would affect not only the subsequent rating session (from E1 to R2 or E2 to R3), as in the classical free-choice paradigm, but also the distant rating session (from E1 to R3). For this purpose, the model was extended to allow action-related biases to accumulate across sessions of the effort task:

$$R_{i}^{k+1}=V_{i}+\sum_{k\prime =1}^{k}b_{i}^{k^{\prime }}+\varepsilon$$ (4)

This equation makes similar predictions to Eq 3 regarding the first two ratings (R1 and R2). However, while Eq 3 assumes that the last rating (R3) is only affected by actions made in E2, Eq 4 now suggests that R3 will also be affected by actions made in E1. As seen before, switching on or off the respective bias parameters corresponding to the four factors (choice, success, force and time) generates 16 models. Among these 16 models, two belong to the null hypothesis H0 (no effect at all or only an effect of time), and 14 represent the hypothesis H2 that actions induce a durable bias that can be expressed in distant ratings.

A family-wise comparison between H0 (2 models), H1 (14 models), and H2 (14 models) sets showed that H2 provided the best explanation of the data (Ef = 0.78, xp = 1), suggesting that actions had a lasting effect on distant ratings. Furthermore, as in previous analyses, grouping models by action-related variables showed that choice and success bias, but not force bias, were significantly present in the population (all xp = 1).

Although this analysis suggests that actions have a lasting effect, it does not imply that this effect would affect other value-driven behaviors than ratings. In other words, the effect could only change declarative judgments, with no influence on subsequent actions and related markers (choice, success, and force).

### Can actions affect behavior beyond declarative judgment?

The question here is to assess whether past actions can influence not just rating but a common hidden value that would drive behavior in both the rating and effort tasks. For this purpose, we needed a quantitative model of how value drives choice, success and force. Inverting this model could then provide evidence that observed choice, success and force data were generated by constant values versus values evolving under the influence of past actions. Following our framework, we modelled all action-related behaviors (choice, success and force) as resulting from a cost/benefit trade-off.

More precisely, we modeled probabilities of choice and success using sigmoid functions, and force using an affine function, of a weighted balance between the cost (required force level L) and the benefit (proposed item value V) involved in every trial. Formally, dropping the indexing of items (i) and repetitions (k) for the sake of readability, we have:

$$\mathrm{choice}\;\mathrm{function}:\;p(C=1)=sig(\rho_{C}V+\eta_{C}L-C_{0})$$ (5)

$$\mathrm{success}\;\mathrm{function}:\;p(S=1)=sig(\rho_{S}V+\eta_{S}L+S_{0})$$ (6)

$$\mathrm{force}\;\mathrm{function}:\;F=\rho_{F}V+\eta_{F}L+F_{0}+\omega$$ (7)

where ω ~ N(0,σ_F_) is some Gaussian noise, ρ_X_, η_X_ and the offsets X_0_ (with X standing for C, S, or F) are free parameters that were estimated through Bayesian inversion. Focusing for example on choice, Eq 5 predicts that the probability of accepting the trial will be higher than average (sig(C_0_)) if ρ_C_V + η_C_L > 0, i.e. if the benefit (item value) is higher than the required cost (force target). As a sanity check, we verified with t-tests that ρ parameters were significantly positive, irrespective of the considered hypothesis (see S1 Table for details). This confirms that values were susceptible to conveying the effects of past actions on subsequent behaviors, since they enhanced the probability of choice and success, and the amount of exerted force.

Critically, in this new set of alternative models, the biases induced by past actions (according to Eq 2) do not impact the ratings directly (as in Eqs 3 or 4), but the underlying values. This is formalized in the following update rule:

$$V^{k+1}=V^{1}+\sum_{k\prime =1}^{k}b^{k^{\prime }}$$ (8)

Where V^k^ is the value of an item (index i has been dropped for readability) during task k, and V^1^ is a free parameter that captures the initial value of this item. The dynamical values (V^k^) were then used to predict not only ratings, using Eq 1, but also choice, success and force, using Eqs 5–7, with V being replaced by V^k^. In fact, the predicted ratings are the same as in the previous set of models, because combining Eqs 1 and 8 is mathematically equivalent to Eq 3. The key difference is therefore the prediction of behavior in the effort task (choice, success and force), which is allowed to vary according to past actions. Again, switching on or off the different bias parameters in Eq 8 yields 16 models: two belonging to the null hypothesis (no bias or time bias only), and 14 to the new hypothesis labeled H3, which implements a two-way causality between values and actions.

We could not compare H3 directly to H1-H2 models as they were formulated in previous sections, because Bayesian model selection can only be performed between models fitted to the same data. Yet previous model comparisons exclusively considered rating data, while the additional prediction brought by H3 is about choice, success and force data. Thus, we rephrased H1 and H2 by including Eqs 5–7, where values were constant since the biases affected not values but ratings (through Eq 3 for H1 and Eq 4 for H2), as schematized in Fig 4. Importantly, these extended H1 and H2 models provide a null hypothesis for the behavior in the effort task: they postulate that past actions have an influence on ratings but not on choice, success and force. Thus, comparing H3 to extended H1-H2 models enables testing for the presence of a bias in the effort task above and beyond chance, properly controlling for potential regression to the mean artifacts.

A family comparison including all models (H0 to H3) showed that indeed H3 best explained the data (Ef = 0.49, xp = 0.98). Post-hoc analyses of the fitted bias parameters in H3 confirmed a positive effect of past choice and success on underlying value, corroborating our previous results, and a non-significant effect of past force. Furthermore, extending models for H1 and H2 did not alter previous conclusions, as significant effects reported in previous sections were still present (see Table 2 for details). We also checked that critical effects captured in our best model (H3) were significant in each experiment analyzed separately, ensuring replicability of our findings across participant groups and task versions (see S2 Table).

**Table 2:** Effects of action-related factors on all subsequent behaviors (rating, choice, success, force) in extended models (H0 to H3).

Family	Factor	Bayesian FFX (xp [Ef])	BMA Estimate (mean±sem)	p-value
H1	Choice (b_C_)	1.00 [0.99]	+ 2.1 ± 0.3	< 0.001 ***
	Success (b_S_)	1.00 [0.63]	+ 0.8 ± 0.1	< 0.001 ***
	Force (b_F_)	0.00 [0.01]	+ 0.0 ± 0.1	0.887
	Time (b_T_)	1.00 [0.99]	– 0.1 ± 0.5	0.800
H2	Choice (b_C_)	1.00 [0.99]	+ 1.8 ± 0.2	< 0.001 ***
	Success (b_S_)	1.00 [0.94]	+ 0.8 ± 0.1	< 0.001 ***
	Force (b_F_)	0.00 [0.12]	+ 0.0 ± 0.1	0.713
	Time (b_T_)	1.00 [0.99]	– 0.3 ± 0.5	0.543
H3	Choice (b_C_)	1.00 [0.99]	+ 1.8 ± 0.2	< 0.001 ***
	Success (b_S_)	0.99 [0.73]	+ 0.7 ± 0.1	< 0.001 ***
	Force (b_F_)	0.00 [0.20]	+ 0.0 ± 0.1	0.758
	Time (b_T_)	1.00 [0.99]	– 0.3 ± 0.5	0.501

Results of group-level random-effect (RFX) Bayesian model selection for each factor and each hypothesis (model space including both H0 and tested hypothesis). Exceedance probability (xp) and expected frequency (Ef) are given for the family of models that includes the considered factor versus all other models. Bayesian Model Average of the corresponding bias (b) parameter is computed for each hypothesis separately and given as mean ± inter-subject SEM. Significance of the respective t-tests against 0 are noted

*** p<0.001, ** p<0.01, * p<0.05. Note that all behavioral variables were fitted here, whereas only ratings were fitted in the BMS reported in Table 1.

**Fig 5:**
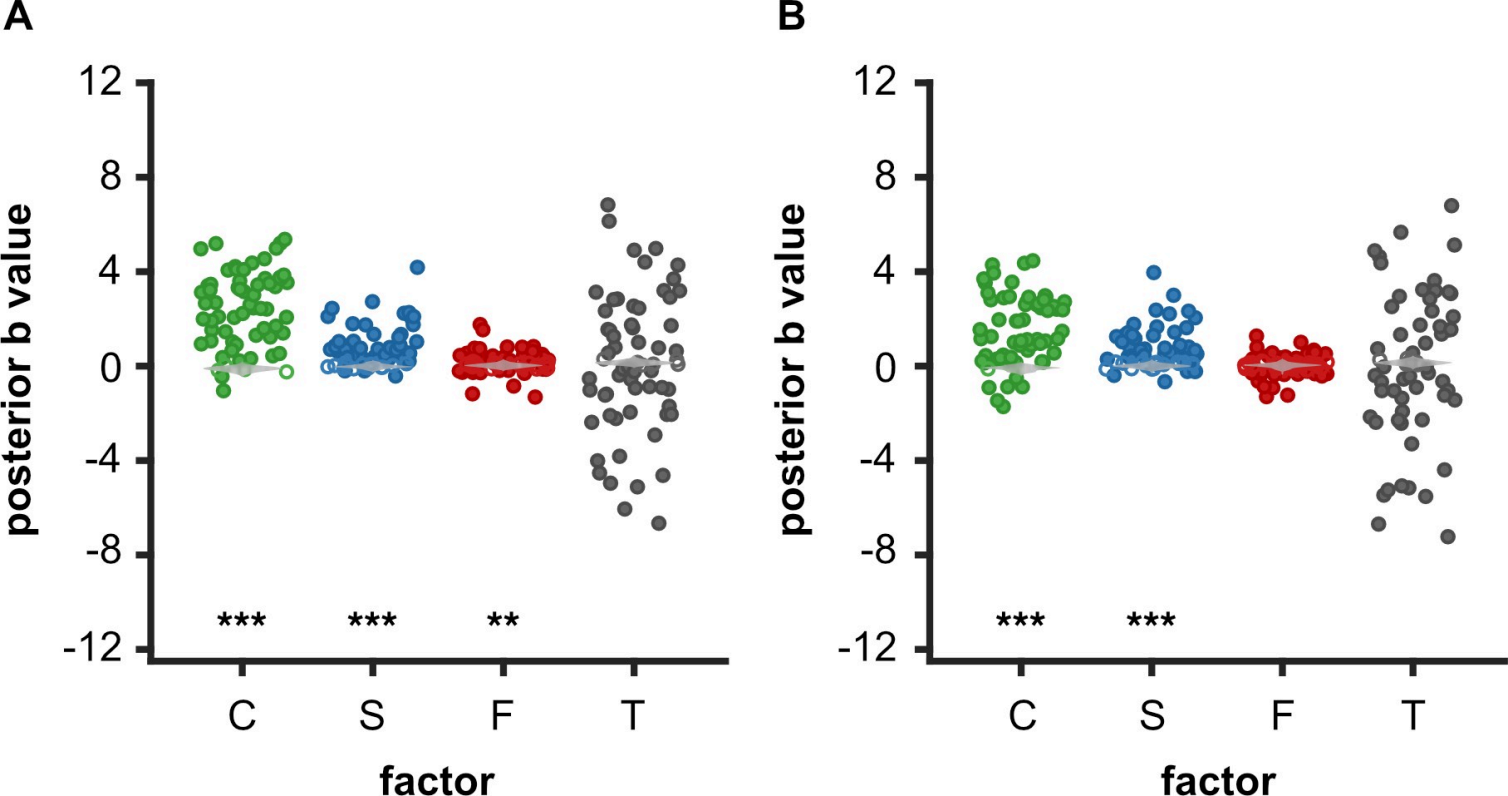
Model-based assessment of whether values are influenced by past actions. Posterior estimates of bias parameters were obtained using (A) BMA on all models (H0-H2) of ratings only (B) BMA on all extended models (H0-H3) of both rating and action-related variables. Bias parameters are the weights assigned to choices (C, green), success (S, blue), force (F, red) and time (T, grey) in equations used to update ratings (H1-H2) or values (H3). Each dot represents one subject. The violins (in light grey) represent the bootstrap distribution of the respective posterior parameters under the null hypothesis (see Supplementary Material). Hollow dots represent subjects for whom the posterior estimate falls within the 95% credible interval of the null distribution. Significant deviation from the null (see Supplementary Material) is indicated as following: ** p<0.01; *** p<0.001.

Finally, we verified that our Bayesian model comparison approach was indeed immune to the ‘regression to the mean’ artifact. For this purpose, we ran Monte-Carlo simulations to a) generate mock behavioral data under the null hypothesis H0 and then b) estimate the effect of past actions in these random data under H1, H2 or H3 (see S1 Text, S3 Table and S4 Table). This resulted in an approximate distribution of the bias parameters (weights of choice, success, force and time) under the null hypothesis (see Fig 5). We then compared these parameter estimates recovered from simulated data to parameters estimated on the data acquired in real subjects. To do so, we compared the 63 real parameters to 63 simulated parameters (randomly selected from the distribution) using Welch’s (unequal variance) two-samples t-tests. This procedure was repeated 1000 times to obtain stable p-values (by averaging over repetitions). These p-values (Fig 5) show that bias parameters estimated in real subjects largely deviated from chance (parameters simulated under H0) for both choice and success, but not force. This was true whether we used only rating data (H1-H2) or both data from both the rating and effort tasks (H1-H2-H3). It confirms that the effects of past actions reported in previous analyses are unlikely to have arisen from a statistical artifact. Moreover, this bootstrap approach also provides for all action-related factors an estimate of the effect size that should be expected from chance (in the absence of a true influence on values), corresponding to the statistical confound pointed by Chen & Risen (see Supplementary Material for details).

Critically, these simulations show that our Bayesian approach did not inflate the amplitude (estimates are always close to zero) nor the significance (false positive rate is kept under nominal threshold) of bias parameters, as it would be the case with classical analyses (see S3 and S4 Tables). More than a mere sanity check, it therefore demonstrates that the statistical method developed in this paper is immune to the artifact identified by Chen & Risen.

## Discussion

In this paper, we examined the possibility of reverse causality from actions to the underlying values that drive these actions. This idea has a long history and has been notably defended in the context of cognitive dissonance theory [7, 8]. We see three major advances in our findings: 1) a novel Bayesian approach discarding the statistical artifacts that had undermined fifty years of research on cognitive dissonance [14, 16], 2) evidence that beyond choice, other action-related variables, such as success, can modulate underlying values, and 3) a demonstration that actions impact not only declarative value judgments but also other value-driven behaviors, such as subsequent decision, effort production and eventual success. These findings are globally consistent with the notion of reverse causality, meaning that the brain would update values based on the behavior which it has itself triggered under environmental constraints [6]. In the following, we successively discuss these findings and their general implications.

The cornerstone of cognitive dissonance theory is the free choice paradigm, which implements a choice-rating-choice series of tasks. The classical result that has been repeatedly observed is that choice seems to affect the change from first to second ratings assigned to the two items. The critique raised by Chen & Risen [14] has casted doubt on this result, which could arise from a regression to the mean artifact. If ratings are considered as noisy projections of hidden values, then two items could get similar first ratings by chance, while second ratings would be closer to the means and reveal a difference that would also be expressed in choice. One experimental solution suggested by Chen & Risen is to add a control condition that implements a rating-rating-choice sequence. This condition enables measuring the statistical artifact in isolation, i.e. without any possible influence of choice on rating, since the choice task is performed after the second rating task. Using this condition as a reference, several authors confirmed a spread of preference beyond any statistical artifact [13, 18, 20, 27], but see [15, 16, 18] for null or mixed results.

To directly address this issue, and rather than introducing a new experimental control condition, we utilized a novel computational strategy that simply compare models with and without an effect of choice on rating. This involves using Bayesian model inversion and comparison techniques to derive the plausibility of an effect beyond what can just be explained by noise in rating and choice behaviors [42–44]. This procedure also provides an estimate of the true effect, and its coherence across subjects, above and beyond (i.e., controlling for) statistical artifacts. In line with classical statistical analyses, we indeed found a choice-induced spread of preference that cannot be reduced to a statistical artifact. This may be an important contribution to the debate but also an important methodological point, since such modeling approach could be applied to any dataset compromised by the same statistical concern.

Although we kept the term choice to underline the link with the existing literature, our choice task was quite different from the typical free choice paradigm. Instead of expressing a preference between two items, participants decided whether or not to exert a given force for a particular food item. The spread of preference was therefore measured between items for which participants made an effortful attempt and the others. We observed no significant impact of the way choice was expressed in the different versions of the paradigm (pressing on accept / decline buttons versus just trying or not to reach the target). This null result suggests that choice does not have to be explicit in order to induce spread of preference. Beyond choice, our task was designed in order to incorporate two candidate effects of actions on values: the so-called ‘sour grape’ and ‘fruit of labor’ effects.

As the grapes in Aesop’s fable, some food items were paired with unreachable force levels, thereby introducing cases of failure. We indeed found that, as the famous fox, our subjects rated items that they failed to obtain as less desirable than initially judged. The usual interpretation for the fox despising what he could not get is that he is trying to avoid regret or humiliation, and maintain the illusion that he is in control. However, following the idea of reverse causality, it could also represent a true change of preference. Indeed success may be a rough proxy for the amount of resources deployed, which should scale with the desirability of the goal. Thus, the brain would logically conclude from observing failure that the goal value is lower than initially estimated. This inference might represent an adaptive mechanism: decreasing the value of unreachable goals could save time and energy. The implication is that updating value would be easier or more efficient than monitoring action feasibility. Yet exploring the environments in which this speculative statement is correct would require comprehensive simulations, using an evolutionary game theoretical approach. Finally, we note that, even if we focus in this discussion on the effect of failure to push the analogy with the sour grapes fable, our model-based analysis does not distinguish between failure and success effect (or accept and decline effects for choice). Thus, all we can conclude is that relative to the initial difference, the difference in desirability between obtained and missed items tends to increase.

Regarding effort expenditure, the reverse causality hypothesis would predict a positive effect of force produced on value, since deploying more resources would signal a more desirable goal. This sort of inference would explain what has been coined ‘fruit of labor’ or ‘effort justification’ effect–the fact that a reward is much appreciated when it comes as an output of strenuous effort [31]. By extension, it could also explain the so-called ‘sunk-cost’ effect [45, 46]–the fact that the propensity to invest resources for attaining a given goal augments with the amount of resources invested in the past with the same goal. Indeed, if the goal value increases with the expended effort, subsequent cost-benefit arbitrage may shift to producing even more effort. However, we found no consistent evidence for produced force to affect subjective value of items, neither with family-wise model nor with classical test on (BMA) posterior estimates. Nonetheless, we believe that this null finding should not be taken as a strong argument against effort affecting value, as it could result from a particular feature of our design. Indeed, as the force produced was mainly driven by imposed target level, it was not so informative about how valuable an item is. It remains possible that an effect may be observed in situations where the outcome would not be binary but proportional to invested effort (as when payoff scales with the amount of work).

More generally, cognitive dissonance theory invokes self-consistency mechanisms to account for the influence of choice on rating. The idea is that agents align their ratings onto their choices because they want to maintain coherence (reduce dissonance), either for themselves or for other people (experimenters). Although this mechanism has long been considered subconscious, it was recently suggested that it might require an explicit memory of the choices made [27, 47]. Here, we did not control for episodic memory effects, so we cannot discard the possibility that our findings represent an explicit adjustment aiming at reducing dissonance between behaviors.

Yet this assumption of explicit adjustments may be tempered by the observation that past actions modulated not only subsequent ratings but also next choice, success and force outputs. Notably, adjusting force just for the sake of showing coherence would entail a deterrent cost. Moreover, keeping in mind all past behaviors, so as to ensure coherence with current behavior, would rapidly become intractable. Updating one value that drives all behaviors appears as a more reasonable strategy, and a more parsimonious explanation. This is even more critical for cognitive dissonance effects that were shown to last for days to years [19, 20]. Value updating could also be an explanation for choice repetition observed in real life, e.g. for supermarket customers aligning their current purchases to their previous brand selection [48]. In the first version of the paradigm, a direct causality from past behavior to next behavior may have strengthened the effects. This was facilitated by the fixed pairing between food items and force levels. In other words, memory of failure could lead to no-try, not because reward value was decreased but because one would expect to fail again. However, such a mechanism would not explain the observed modulation on subsequent rating. Moreover, we observe a similar spread of preference in subsequent versions of the paradigm, which proposed different pairings between food items and force targets in the two effort tasks. So we conclude that reverse causality from actions to values, with values then driving subsequent behaviors, is a more parsimonious account for our set of observations. This mechanism may be seen as a shortcut implemented by the brain to avoid repeating bad choices and experiencing failure.

However, there are a number of limitations in our study. A first limitation is that we did not consider the possibility that overt ratings may be processed similarly to other actions, eventually resulting in some sort of rating-induced value changes. This was omitted for the sake of simplicity but in principle, people could get information about their values from their own statements. A second limitation is that in our design, choice was always expressed actively. Indeed, to accept an offer, participants had to lightly squeeze the grip (in Exp 1) or to press a button (in Exp 2 and 3). In other words, we did not include a "no-go" condition in which acceptance would be indicated by inaction. Different results might have been obtained in this condition, since it has been shown that positive and negative items are revaluated differently depending on whether choice is active or inactive [21], possibly due to the implication of dopaminergic signaling in linking action to reward [49]. A third limitation is that we did not specify the computational mechanism used to update values. In our update equation, action variables have linearly cumulative effects, again for the sake of simplicity. This was sufficient to afford statistical evidence for the effect of interest but may be deemed unrealistic, because such a linear accumulation would eventually yield diverging values when exposed to repetitive choice and success (something like ‘the more I choose it the more I like it’, then ‘the more I like it the more I choose it’, and so on). Exploring the dynamics of this iterative logic would be way beyond the scope of this paper, since we only have two repetitions in our design.

One natural solution would be Bayesian updating: value should be updated according to (i) its precision: the higher the confidence associated to the value of an item, the less susceptible to change it should be, (2) the prediction error: the influence of a choice should be lower if it was strongly predicted by value. Note that choice and success are binary (0 or 1), so they necessarily differ from their prior probability. Consistent with the latter principle, it has been shown with the free choice paradigm that easy choices (i.e., choices that have prior probability close to 1, because of a high difference between option values) have a lesser influence on rating change than hard choices [16]. The former principle would ensure convergence, since precision would increase after choice, stabilizing values even if choice (and success or failure) were repeated over and over.

To make sense, such a model would need to assume some encapsulation of the brain systems that generate action and those that update value. If the latter system were fully informed about the computations performed by the former system, there would be no discrepancy between observed and predicted behavior, and therefore no possibility of learning. With two separate systems, one could learn from the other: the value-updating system would integrate a rough initial prediction from a superficial inspection of the choice situation, and then learn from the discrepancy between that prediction and the eventual behavioral response generated by the action-selection system after a more careful consideration.

In summary, we have provided a computational method to test the effects of actions on underlying values while properly controlling for statistical artifacts. Using this method, we have extended the classical effect of choice on likeability judgment, showing that not only choice but also experience of success versus failure may modulate the hidden values that in turn impact not only preference but also subsequent effort expenditure and eventual outcomes. Altogether, this work generalizes the notion of a two-way dynamic interaction between actions and values, which may invite a serious reconsideration of decision theory.

## Methods

### Ethics statement

The research has been approved by the Ethics Committee for Biomedical Research (‘Comité de Protection des Personnes’) of the Pitié-Salpêtrière Hospital. All participants gave written informed consent prior to participating in the study.

### Participants

Three groups of participants (n: 18, 24, 24; gender: 13/5, 17/7, 15/9 female/male; age±SEM: 23± 0.7, 24±0.9, 24±0.8 correspondingly) were recruited from a volunteer database in Paris to participate in experiments 1 to 3. They were asked to refrain from eating at least 3 hours before arriving in the lab, but were allowed to drink water (sugary drinks were not allowed). On average their last meal was 5h43±51min, 6h18mn±42min and 5h48±39min before the start of the study. They received 30 euros for their participation and up to two food products, corresponding to the two trials that were drawn at random at the end of the two effort tasks. Before the experiment, participants were shown a large subset of the potential food prizes stored in the testing room. They were then informed that they could only get the food items that they successfully obtained during the task, and that there would be a lottery to select the two trials that determined their food reward. Three participants were excluded because they had too few failures (they almost always succeeded after accepting a trial), meaning that the effects of choice and success were not separable. The analysis was therefore conducted on a total of 63 participants.

### Stimuli and apparatus

We used 150 standardized images of sweet and savory snack products available in French supermarkets (106 taken from the French INSEAD food database, and 44 were created in the lab). Food products were photographed frontally over a black background, with some of the contents displayed in front of the packaging. Size of the photographs was 400x300 pixels. All experimental stimuli were presented via MATLAB (www.mathworks.com) and Psychtoolbox (http://psychtoolbox.org [50]). The experiment was run on a Windows-based PC.

A handmade pneumatic handgrip device was used in Exp 1 (as in Ref 34), whereas Vernier Hand Dynamometers (https://www.vernier.com) were used in Exp 2–3.

### Behavioral tasks

Three versions of the behavioral paradigm were designed for the three experiments.

#### Version 1 (Exp 1)

For force calibration, we separately measured the maximum force level (F_max_) of each hand as participants squeezed the grip as strongly as possible for 3s. This subject-specific, hand-specific measure was used to calibrate target force levels in effort task trials. Subsequently, participants performed a series of two tasks: rating (R) and effort (E) tasks alternated following a R1-E1-R2-E2-R3 order. During the rating task, participants judged the desirability of all food products. During the effort task, participants were presented with a food item paired with a target force level and had to make squeeze/no squeeze decision to earn or reject the item. Participants were not told in advance that these tasks would be repeated. In all tasks, participants responded at their own pace.

Rating task (120 trials). Participants were shown an image of a food item indicated on a visual analogue scale (VAS) the extent to which they desired to eat the food item (Fig 1A). They were instructed to imagine having the real item in front of them, opening the packaging, smelling the odor, and tasting it in their mouth. They then used the keyboard to move the cursor and indicated their desire to eat the item at that precise moment. Participants knew how long the task would take approximately and the number of items to rate.

Effort task (90 trials). Based on subject-specific ratings in the first session (R1), we excluded the 15 lowest and 15 highest ranked products and used the remaining in the effort task. Each item was paired with a given target level of force, between 20% and 120% F_max_. To ensure that target force level and desirability rating were uncorrelated, we formed a diamond-shaped pairing (see Fig 1C), such that the lowest and highest ranked items were paired with levels around 70% F_max_ (i.e., midpoint between 20% and 120%) whilst the middle-ranked items were paired with levels around 20% or 120% F_max_. Furthermore, we paired adjacent ranked items with force levels symmetrically spaced above and below the midpoint. The effort-rating pairing was the same for the first and second effort task (E1 and E2), but the order of item presentation was randomized within each effort session and for each participant.

The critical outcomes in the effort task are choice, success, and amount of exerted force. Each trial began with 1s acquisition of force baseline levels, during which participants were told not to squeeze, and 1s fixation cross. Participants were then presented with one food picture at the center of the screen and an image of an empty thermometer next to it (with display side counterbalanced across subjects). At this time, the target force level was not indicated, but participants could disclose it by lightly squeezing the handgrip (5% F_max_). Squeezing the grip moved the red bar that served as a visual feedback on the force produced. Participants had the opportunity to earn the food item by maintaining the bar above the target force level for at least 4.5s consecutively. Success was confirmed by a green frame around the food item for 2s. At any point, participants could press a 'skip' key on the keyboard using their non-squeezing hand to reject the food item, in which case the item was crossed out but remained on screen for 2s. Inter-trial interval was 1-2s, uniformly distributed.

In addition to the force produced and the eventual skip or success outcome, we defined choice as the attempt to obtain the outcome: the trial was considered as accepted if the participant produced the minimal target force level (20%*F*_*max*_). Conversely, the trial was considered as rejected if the participant pressed the skip button without producing even this minimal force. The other behavioral variables, success and force, were defined only for accepted and successful trials respectively, in the aim of decorrelating them from the choice variable.

In order to reduce potential fatigue effects, participants switched hands every 15 trials. They all started with the right hand. Before the start of each effort task session (E1 and E2), participants understood that they would receive one food item randomly selected amongst those with which the target force levels were successfully reached. In total participants received two items, one from each of the two effort tasks.

#### Version 2 (Exp 2)

Exp 2 was very similar to Exp 1, except for the following changes.

- In the rating task, we used 150 items instead of 120, leading to 150 rating trials.
- In the effort task (90 trials), several changes were made. (1) We randomly selected 30 food items from the rating task to be excluded, in addition to the 15 lowest and 15 highest ranked products. The goal was to estimate how declarative judgments of desirability may drift, independently from any choice or effort production. (2) Different pairings between target force level and desirability rating were used in the two sessions of the effort task (E1 and E2). The goal was to avoid repetition effects between E1 and E2 and to ensure that participants would evaluate the cost/benefit tradeoff even in the second session. Therefore, for each participant, items and targets were pseudo-randomly paired so as to orthogonalize not only force level and desirability rating but also force levels used in E1 and E2 for the same item. (3) In order to reduce fatigue, the minimal above-target duration required to earn the item was 3s in Exp 2 (instead of 4.5s in Exp1). (4) The action required to disclose target force level was pressing a ‘yes’ button in Exp 2 instead of a light squeeze on the handgrip (5% *F*_*max*_) in Exp1. Alternatively, pressing the ‘no’ button allowed declining the offer and proceeding directly to the next trial (without seeing the target force level). This change was implemented in order to avoid the possibility that participants accidently pass the 20% *F*_*max*_ threshold while they only intended to disclose target force level (corresponding to 5% *F*_*max*_). (5) Finally, before each effort task session, participants were informed in Exp 2 that one trial would be randomly selected and realized, meaning that they would either get the food item or nothing depending on whether or not they successfully reached the target force level. This payoff schedule was different from Exp 1, where one successful trial was randomly selected and realized, leaving participants the liberty to decline all food items but the one they prefer. Fortunately, none of the participants employed this risky strategy, even in Exp 1 (acceptance rate was around 50% in all 3 experiments).

#### Version 3 (Exp 3)

Exp 3 was similar to Exp 2 except for further changes in the effort task. (1) Both food item and target force level were presented, simultaneously, at the beginning of the trial. Then participants could accept or reject the offer, by pressing either the ‘yes’ or ‘no’ button. Thus, acceptance was indicated explicitly, compared to the light squeeze criterion (>20%*F*_*max*_). This explicit commitment made a difference: the rate of failure (trials in which participants did try but failed to reach target force) was lower is this version of the task compared to the two first versions. Despite the low number of data points, we did observe significant success effects on subsequent ratings in this last version. However, it might not be ideal to investigate the sour-grape effect, because the statistical power of comparison between success and failure was quite weak.

### Analyses

All analyses were run with Matlab (www.mathworks.com). Computational modeling, estimation, and simulation, as well as follow-up Bayesian analyses [40] were implemented using the VBA toolbox [42] (http://mbb-team.github.io/VBA-toolbox/). Regression coefficients are given as mean + sem. Goodness of fit is the balanced accuracy for logistic regression (choice, success) and R2 for linear regression (force), also given as mean + sem. All t-tests are two-tailed. Details on computational models are provided in the results section.

## Supporting information

S1 Text Bootstrap validation. (DOCX)

S1 Table Weights of hidden values on action-related factors. (DOCX)

S2 Table Effects of action-related factors on all subsequent behaviors (rating, choice, success, force) in extended models (H3), separately for each experiment. (DOCX)

S3 Table Summary of the bootstrap analysis for extended models. (DOCX)

S4 Table Summary of the bootstrap analysis for restricted models. (DOCX)
