# Supplementary material for: Sour grapes and sweet victories: How actions shape preferences
Source: PLoS Comput Biol. 2019 Jan 7;15(1):e1006499. doi: 10.1371/journal.pcbi.1006499 (PMC6344105; doi:10.1371/journal.pcbi.1006499)
Supplement: S1 Text — (DOCX) [file pcbi.1006499.s001.docx]

Sour grapes and sweet victories: how actions shape preferences

Fabien Vinckier*, Lionel Rigoux*, Irma T. Kurniawan*, Chen Hu, Sacha Bourgeois-Gironde, Jean Daunizeau, Mathias Pessiglione

# Supplementary Results: Bootstrap validation

One potential concern the reader might have about our computational approach is the validity of Bayesian model inversion techniques to address - and completely correct for – the statistical issue raised by Chen & Risen. We thus performed Monte-Carlo simulations to evaluate the soundness of our approach, i.e. its ability to capture the effect of past actions (bias parameters) on subsequent behaviors, without inflating the effect size as seen with classical statistics.

To do so, we first simulated synthetic data under the null hypothesis. More precisely, we first computed for each subject the Bayesian model average of all parameters and hyperparameters. We then collated those estimates across individuals to obtain for every parameter and hyperparameter a unique distribution that fully described the computational profile of the population. If we now draw a random set of parameters from this distribution and plug them back in our model, we are able to generate synthetic data that are similar (i.e., with the same statistical properties) to the real observations made in our subjects. We simulated n=100 of those virtual subjects, to one notable exception: we switched all bias parameters (*b*) to zero. We finally formed N=1000 synthetic groups by randomly picking subjects, 18 at a time, in the synthetic population. Any of these control groups forms a counterpart in all points similar to our genuine test sample but for the fact that they display strictly no effects of past actions (i.e., they follow H0).

Then, we applied to each of the simulated dataset the same analysis (model inversion, family-wise hypothesis selection, model averaging and t-test on posterior parameters) that we used for real data in the main text. Critically, this procedure provides a confidence interval on the summary statistics (p-values, exceedance probability) knowing there is no bias in the behavior. In other words, we are able to predict the probability of mistakenly concluding for the presence of a bias (false positive error rate).

All results are summarized in the table below (Supplementary Table 3 and 4). The posterior means of all bias parameters were close to zero, showing the estimation was not distorted by the regression to the mean artifact, and much smaller than those observed in real subjects. Accordingly, *t*-tests against zero gave large p-values (around 0.5), and the probability of rejecting the null (ie. to conclude for the existence of an effect) did not pass under the nominal significance threshold (0.05) more often than expected at chance (i.e., 5% of the time). Family comparison always favored the null hypothesis (H0) and consistently rejected all potential effects of past actions (H1-H2 for models of ratings only, H1-H3 for models extended to all behaviors). Importantly, the exceedance probability of biased models (H1-H3) fitted on those simulated data never reached the level of significance that we observed in our experimental group.

We then tested whether the posterior parameters that we observed in our real sample systematically deviated from the bias parameters estimated in our synthetic control groups. To do so, for each of the 1000 synthetic samples, we compared the two groups by applying two samples (unequal variance) Welch's *t*-tests on every bias parameter. The computed *p*-values were then averaged across the Monte-Carlo simulations. As expected (see Supplementary Table 3), this procedure substantiated, in extended models, an effect of choice (*p*=0.003) and success (*p*<0.001), but no consistent effects of force (*p*=0.297) or time (*p*=0.078). The same analysis performed on the reduced models (rating only) gave qualitatively identical results (Supplementary Table 4).

Altogether, those simulation results validate our working premise that our Bayesian modeling approach is immune to the statistical bias that flaws classical statistic approaches. Unlike statistical methods used in previous studies, our method does not suffer from an inflated false positive rate.
