# Supplementary material for: Sour grapes and sweet victories: How actions shape preferences
Source: PLoS Comput Biol. 2019 Jan 7;15(1):e1006499. doi: 10.1371/journal.pcbi.1006499 (PMC6344105; doi:10.1371/journal.pcbi.1006499)
Supplement: S1 Table — (DOCX) [file pcbi.1006499.s002.docx]

Sour grapes and sweet victories: how actions shape preferences

Fabien Vinckier*, Lionel Rigoux*, Irma T. Kurniawan*, Chen Hu, Sacha Bourgeois-Gironde, Jean Daunizeau, Mathias Pessiglione

# Supplementary Results

**Weights of hidden values on action-related factors.**

As a sanity check, we estimated the amplitude of the impact that hidden values had on each action-related variable (choice, success and force produced) by computing the Bayesian Model Average of the posterior weight $\rho$ and entered them into group-level random-effect analyses. As summarized in Table 1, $\rho_{C}$, $\rho_{S}$, and $\rho_{F}$ were significantly positive, whichever the considered model family. This is consistent with the idea that updated hidden values drive all action-related variables.

|  | Choice($\boldsymbol{\rho}_{\boldsymbol{C}}$) | Success($\boldsymbol{\rho}_{\boldsymbol{S}}$) | Force($\boldsymbol{\rho}_{\boldsymbol{F}}$) |
| --- | --- | --- | --- |
| H1 | + 6.4 (0.2) *** | + 2.0 (0.2) *** | + 0.06 (0.02) *** |
| H2 | + 6.5 (0.2) *** | + 2.0 (0.2) *** | + 0.06 (0.01) *** |
| H3 | + 6.5 (0.2) *** | + 2.0 (0.2) *** | + 0.06 (0.01) *** |

Supplementary Table 1: Weights of hidden values on action-related variables.

Bayesian Model Average of the corresponding weight ($\rho_{X}$) parameter is computed for each hypothesis separately and given as mean ± inter-subject SEM. *** p<0.001, ** p<0.01, * p<0.05
