# Supplementary material for: Sour grapes and sweet victories: How actions shape preferences
Source: PLoS Comput Biol. 2019 Jan 7;15(1):e1006499. doi: 10.1371/journal.pcbi.1006499 (PMC6344105; doi:10.1371/journal.pcbi.1006499)
Supplement: S2 Table — (DOCX) [file pcbi.1006499.s003.docx]

Sour grapes and sweet victories: how actions shape preferences

Fabien Vinckier*, Lionel Rigoux*, Irma T. Kurniawan*, Chen Hu, Sacha Bourgeois-Gironde, Jean Daunizeau, Mathias Pessiglione

# Supplementary Results

**Effects of action-related factors on all subsequent behaviors (rating, choice, success, force) in extended models (H3), separately for each experiment.**

In order to ensure that critical effects were replicable, the best model (H3) was fitted separately for each experiment (Exp 1-3). The influence of choice and success, but not that of force and time, was significant in all 3 experiments.

| **Experiment** | **Factor** | **Bayesian FFX (xp [Ef])** | **BMA Estimate (mean±sem)** | **p-value** |
| --- | --- | --- | --- | --- |
| 1 | Choice (*b*_C_) | 1.00 [0.97] | + 1.4 ± 0.5 | 0.008 ** |
|  | Success (*b*_S_) | 1.00 [0.97] | + 1.0 ± 0.2 | < 0.001 *** |
|  | Force (*b*_F_) | 0.00 [0.01] | – 0.4 ± 0.2 | 0.098 |
|  | Time (*b*_T_) | 1.00 [0.97] | – 1.6 ± 1.1 | 0.166 |
| 2 | Choice (*b*_C_) | 1.00 [0.98] | + 1.8 ± 0.4 | < 0.001 *** |
|  | Success (*b*_S_) | 1.00 [0.98] | + 0.5 ± 0.2 | 0.008 ** |
|  | Force (*b*_F_) | 0.00 [0.05] | + 0.2 ± 0.2 | 0.254 |
|  | Time (*b*_T_) | 1.00 [0.98] | + 0.5 ± 0.5 | 0.327 |
| 3 | Choice (*b*_C_) | 1.00 [0.98] | + 2.0 ± 0.4 | < 0.001 *** |
|  | Success (*b*_S_) | 0.00 [0.11] | + 0.8 ± 0.3 | 0.02 * |
|  | Force (*b*_F_) | 0.00 [0.11] | + 0.2 ± 0.2 | 0.256 |
|  | Time (*b*_T_) | 1.00 [0.98] | – 0.2 ± 0.9 | 0.793 |

Supplementary Table 2: Effects of action-related factors on all subsequent behaviors (rating, choice, success, force) in extended models (H3), separately for each experiment.

Results of group-level random-effect (RFX) Bayesian model selection (within a model space including both H0 and H1) are shown for the three action-related factors in different lines. Exceedance probability (xp) and expected frequency (Ef) are given for the family of models that includes the considered factor versus all other models. Bayesian Model Average of the corresponding bias (b) parameter is given as mean ± inter-subject SEM. Significance of the respective t-tests against 0 are noted: *** p<0.001, ** p<0.01, * p<0.05
