# Supplementary material for: Sour grapes and sweet victories: How actions shape preferences
Source: PLoS Comput Biol. 2019 Jan 7;15(1):e1006499. doi: 10.1371/journal.pcbi.1006499 (PMC6344105; doi:10.1371/journal.pcbi.1006499)
Supplement: S3 Table — (DOCX) [file pcbi.1006499.s004.docx]

Sour grapes and sweet victories: how actions shape preferences

Fabien Vinckier*, Lionel Rigoux*, Irma T. Kurniawan*, Chen Hu, Sacha Bourgeois-Gironde, Jean Daunizeau, Mathias Pessiglione

# Supplementary Results

**Summary of the bootstrap analysis for extended models**

| **Factor** | **Bayesian RFX** | | **BMA estimate (mean±std)** | **p-value (mean±std)** | **P(p<0.05)** |
| --- | --- | --- | --- | --- | --- |
|  | **xp (±std)** | **[EF(±std)]** |  |  |  |
| Any | 0.00 (0.00) | 0.14 (0.00) |  |  |  |
| Choice | 0.01 (0.06) | 0.10 (0.10) | -0.062 (0.141) | 0.48 (0.29) | 0.05 |
| Success | 0.06 (0.20) | 0.13 (0.17) | -0.108 (0.172) | 0.47 (0.28) | 0.05 |
| Force | 0.04 (0.17) | 0.10 (0.15) | +0.001 (0.171) | 0.53 (0.28) | 0.03 |
| Time | 0.18 (0.20) | 0.32 (0.17) | +0.056 (0.070) | 0.44 (0.29) | 0.06 |

# Supplementary Table 3: Summary of the bootstrap analysis for extended models (fitting rating, choice, success and force).

# Posteriors of bias parameters corresponding to the different factors are expressed as means ± std computed across the N=1000 runs of null hypothesis simulations. For each factor, the bootstrapping of the t-test against zero is reported both as the mean ± std of the p-value and as the empirical false positive rate P(p<0.05). Bootstrapped exceedance probability (xp) and expected frequency (Ef) are given for the family of models that include the considered factor against the family of models which do not. The first line "any factor" corresponds to the test of H1-H3 against H0.
